# Diverse Roles of Macrophages in Atherosclerosis: From Inflammatory Biology to Biomarker Discovery

**DOI:** 10.1155/2012/693083

**Published:** 2012-04-11

**Authors:** Ting Gui, Aiko Shimokado, Yujing Sun, Takashi Akasaka, Yasuteru Muragaki

**Affiliations:** ^1^First Department of Pathology, School of Medicine, Wakayama Medical University, 811-1 Kimiidera, Wakayama 641-0012, Japan; ^2^Department of Cardiovascular Medicine, School of Medicine, Wakayama Medical University, 811-1 Kimiidera, Wakayama 641-0012, Japan

## Abstract

Cardiovascular disease, a leading cause of mortality in developed countries, is mainly caused by atherosclerosis, a chronic inflammatory disease. Macrophages, which differentiate from monocytes that are recruited from the blood, account for the majority of leukocytes in atherosclerotic plaques. Apoptosis and the suppressed clearance of apoptotic macrophages (efferocytosis) are associated with vulnerable plaques that are prone to rupture, leading to thrombosis. Based on the central functions of macrophages in atherogenesis, cytokines, chemokines, enzymes, or microRNAs related to or produced by macrophages have become important clinical prognostic or diagnostic biomarkers. This paper discusses the impact of monocyte-derived macrophages in early atherogenesis and advanced disease. The role and possible future development of macrophage inflammatory biomarkers are also described.

## 1. Introduction

Cardiovascular disease (CVD) is the leading cause of mortality in developed countries and is likely to attain this status worldwide, accounting for 16.7 million deaths each year [1, 2]. Coronary artery disease (CAD) and cerebrovascular disease are the most common forms of CVD, whose underlying pathological feature is atherosclerosis. Atherosclerosis is a slowly progressing chronic disease of large and medium-sized arteries which is characterised by the formation of atherosclerotic plaques consisting of necrotic cores, calcified regions, accumulated modified lipids, migrated smooth muscle cells (SMCs), foam cells, endothelial cells (ECs), and leukocytes [3].

 Since the term arteriosclerosis was first introduced by Jean Lobstein in 1829 [4], it has long been believed that atherosclerosis involved the merely passive accumulation of cholesterol in arterial walls. In the 1970s, the response-to-injury model was described [5]. Today, the picture of atherosclerosis is much more complex as it has been considered a chronic inflammatory disease, involving both the innate and adaptive immune systems, which modulate the initiation and progression of the lesions, and potentially devastating thrombotic complications [6]. Understanding the principles of the inflammatory processes is important for deciphering the complex processes involved in atherosclerosis progression. Atherosclerotic plaques are characterised by an accumulation of lipids in arterial walls together with infiltration of immunocytes. The degree of influx of inflammatory cells to atherosclerotic lesions is determined based on monocyte recruitment, macrophage egress, and the balance of proliferation, survival, and apoptosis within the arterial walls [7].

Macrophages are the first inflammatory cells to invade atherosclerotic lesions, and they are the main component of atherosclerotic plaques [8]. Inflammatory cytokines produced by macrophages stimulate the generation of endothelial adhesion molecules, proteases, and other mediators, which may enter systemic circulation in soluble forms [9]. Cytokines as inflammatory biomarkers, independent of cholesterol and regulators of blood pressure, could yield more information on different aspects of pathogenesis of atherosclerosis [10]. This paper discusses the central roles of macrophages in every stage of atherosclerosis, focusing on the role of inflammatory biomarkers in predicting primary cardiovascular events related to macrophages.

## 2. Initiation and Early Progression of Atherosclerosis

### 2.1. Recruitment and Entry of Monocytes to Arterial Walls

**Figure 1:**
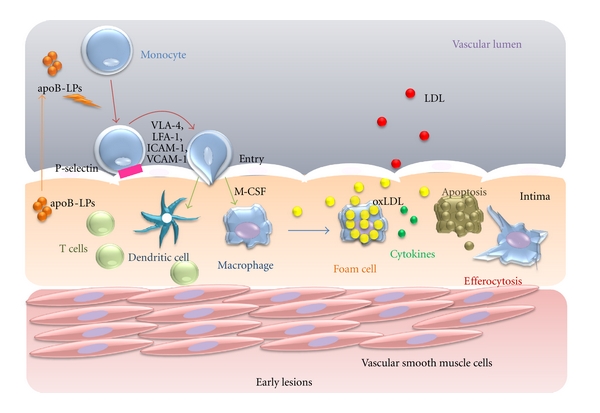
The roles of M1 and M2 macrophages. Ly6C high monocytes differentiate into M1 type, classically activated macrophages that affect proteolysis and produce antibacterial products. Ly6C low monocytes differentiate into M2 type, alternatively activated macrophages that are involved in wound repair and tissue remodelling. M1 and M2 cells secrete different cytokines that function in efferocytosis and the formation of foam cells.

 Monocytes originate from bone marrow-derived progenitor cells and do not proliferate in the blood [11]; their functions under homeostatic conditions remain unclear. The mechanisms of monocyte homing to healthy aortas are not well defined; more is known about monocyte recruitment into aortas during atherogenesis [12]. During the pathogenesis of atherosclerosis, blood monocytes infiltrate from blood to the intima and subintima [13], a process which is activated by subendothelial accumulation of apolipoprotein B-containing lipoproteins (apoB-LPs) [14]. Summoned by chemokinesis, monocytes roll over and become tethered to endothelial cells overlying retained apoB-LPs through interactions between monocyte P-selectin glycoprotein ligand-1 (PSGL-1) and endothelial selectins [14]. E-selectin overlaps with P-selectin to support rolling [15]. After monocytes roll on the inflamed aortic endothelium, they use lymphocyte function-associated antigen-1 (LFA-1), very late antigen-4 (VLA-4) and their respective endothelial cell ligands, including vascular cell adhesion molecule (VCAM-1) and intercellular adhesion molecule-1 (ICAM-1), to slow rolling and form tighter adhesions [16]. Finally, firm adhesion is followed by entry of monocytes into the subendothelial space (diapedesis) [17] (Figure 1).

**Figure 2:**
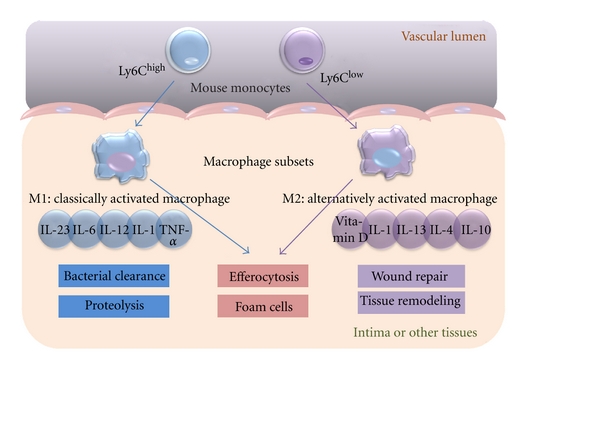
Signalling pathways in a macrophage involved in atherosclerosis. Pro- and anti-inflammatory factors act on macrophages, leading to activation of downstream scavenger receptors (SRs)/toll-like receptors (TLRs)-NF-*κ*B signalling, endoplasmic reticulum (ER) stress and efflux of cholesterol via ABCA and ABCG transporters.

 In mice, monocytes can be identified from other circulating cells by the differential expression of chemokine C-C motif receptors 2 (CCR2), chemokine C-X3-C motif receptor 1 (CX3CR1), and Ly6C antigen, which is monocyte/macrophage cell differentiation antigen regulated by interferon gamma [11]. Apolipoprotein E–/– (*Apoe*–/–) mice, a model system for atherosclerosis, are prone to develop atherosclerosis because they have high levels of the atherogenic lipoprotein known as remnant lipoprotein [18]. Ly6C^high^CCR2^+^CX3CR1^Low^ monocytes, which are precursors of inflammatory macrophages, have been observed to adhere to activated endothelium in* Apoe*–/– mice [19]. In contrast, little is known about how a lack of apoE affects inflammatory Ly6C^low^CCR2^−^CX3CR1^high^ monocytes [20]. These studies suggest that there is persistent recruitment of inflammatory monocytes into established atherosclerotic lesions (Figure 2). These studies described above are limited in mice and it may be difficult to interpret human macrophage subsets, but two major subsets of human macrophages can be defined: CD14^high^CD16^low^ macrophages typically represent 85% ~ 95% monocytes in healthy individuals; CD14^low^CD16^high^ macrophages are comprised in the remains [21]. The role of each subset in human atherosclerosis remains unknown.

### 2.2. Monocyte Differentiation into M1 and M2 Subsets of Macrophages

Driven by macrophage colony-stimulating factor (M-CSF) and other differentiation factors, monocytes differentiate into two major types of macrophages and/or dendritic cells [22, 23]. M1 and M2 macrophages play opposite roles during inflammation, although both are present in atherosclerotic lesions. M1 macrophages, which are differentiated from Ly6C^high^ monocytes and promote inflammation, are classically activated by lipopolysaccharide in the presence of IFN-*γ*, leading to the production of high levels of IL-2, IL-23, IL-6, IL-1, and TNF-*α*. In contrast, activated M2 macrophages, which are differentiated from Ly6C^low^ monocytes and promote resolution inflammation, differentiate in the presence of IL-4, IL-13, IL-1, or vitamin D3 and tend to produce a large amount of IL-10 and express scavenger receptors, mannose receptors, and arginase [24] (Figure 2). Recently, there has been a great deal of interest in macrophage heterogeneity in atherosclerotic lesions, particularly regarding the roles of M1 versus M2 macrophages. There is evidence that an imbalance in the ratio of classically activated M1 and alternatively activated M2 macrophages in advanced atherosclerosis impair resolution *in vitro* [25], but a clear picture has not yet emerged from these studies [23]. Most of the hypotheses in this area have been driven by* in vitro* studies exploring gene expression patterns and functional attributes of monocytes or macrophages subjected to various treatments, including growth factors, cytokines derived from helper T cells [26], the transcription factors peroxisome proliferators-activated receptors (PPARs) *γ* [27], and the bioactive lipid sphingosine-1-phosphate [28]. However, there is a significant difference between *in vitro* and *in vivo* results, which makes atherogenesis more complex. Future projects should focus on the characterisation of macrophage heterogeneity with respect to differential expression of specific molecular biomarkers that have functional significance for atherogenesis [29]. Additional attention should be paid to the roles of cytokines in controlling monocytes that differentiate into dendritic cells (DCs) rather than macrophages.

### 2.3. Important Receptors and Transporters for Cholesterol Loading and Efflux in the Toll-Like Receptors of Macrophages

**Figure 3:**
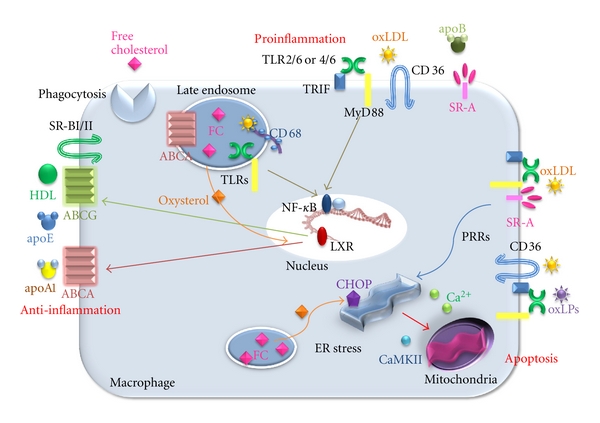
The fate of macrophages in an early lesion of atherosclerosis. The accumulation of apolipoprotein B-lipoproteins (apoB-LPs) in the matrix beneath the endothelial cell layer leads to the recruitment of monocytes. The cells differentiating into macrophages undergo foam cell formation, leading to apoptosis. Because efferocytosis works efficiently, this lesion does not develop necrotic core. The resolution of the inflammation results in decreased plaque progression.

 In the innate immune system, toll-like receptors (TLRs) are the primary receptors that recognise highly conserved structural motifs of pathogens [30]. Under hyperlipidemic conditions, TLRs likely participate in the regulation of atherosclerosis. The activation of TLRs induces the production of proinflammatory cytokines and nitric oxide in macrophages and the induction of DC maturation, leading to the upregulation of costimulatory molecules, such as CD80 and CD86. In addition, TLR1, TLR2, TLR4, and TLR6 are expressed in atherosclerotic lesions. A large number of pathogen-associated molecules can activate TLRs. Heat-shock proteins (hsp60) [31] and oxidised (ox) LDL [32] mediate at least a part of their effects within atherosclerotic plaques through TLR4 binding. TLR2, expressed on cells that do not derive from bone marrow, appears to promote atherogenesis in mice [33]. Interestingly, Sun et al. showed that free cholesterol (FC) accumulation in the endosomal compartment increases the inflammatory response in a TLR-dependent fashion, and TLR3 is the predominant receptor involved in this process [34] (Figure 3).

### 2.4. Scavenger Receptors

Macrophage scavenger receptors (SRs) are found to bind and internalise modified forms of LDL through mechanisms that are not inhibited by cellular cholesterol content, and they are likely responsible for macrophage cholesterol accumulation [35]. SR class A (SR-AI and AII), expressed on the surface of macrophages, account for the uptake of acetylated LDL in the majority of macrophages, but macrophages preferentially bind oxLDL, recognising the modified apoB components of the particles [36]. Interestingly, SR-As expression is increased in animals with low atherosclerotic responses, suggesting that this pathway is protective. Furthermore, overexpressing a secreted form of the extracellular domain of human SR-A resulted in a 20% reduction in monocyte/macrophage adherence to endothelial cells in atherosclerotic lesions in *Ldlr*–/– mice [37]. Thus, the use of such decoy SRs may prove beneficial for retarding the development of early atherosclerotic lesions (Figure 3).

 Other studies indicate that SR class B CD36 plays a major role in the clearance of oxLDL, contributing 60% to 70% of cholesterol ester accumulation in macrophages exposed to LDL oxidised by Cu^+2^ and myeloperoxidase/peroxynitrite [38, 39]. CD36 activates signalling via TLR2 and TLR6 in response to lipoteichoic acid and diacylated macrophage-activity lipopeptide 2 [40, 41]. In addition, a newly described TLR heterodimer of TLR4/6 has been shown to cooperate with CD36 in activating NF-*κ*B in response to oxLDL [42] (Figure 3).

 SR-BI and SR-BII share 30% sequence homology with CD36 and both can bind modified forms of LDL as well as native HDL, LDL, and VLDL [43] (Figure 3). These receptors have a major impact on lipoprotein metabolism through two mechanisms: (1) SR-BI mediates cholesterol transfer to HDL, and (2) SR-BI facilitates selective delivery of lipoproteins from HDL to steroidogenic tissues for excretion into bile and feces in the liver [35]. Although the antiatherogenic effects of SR-BI have been largely attributed to mediation of cholesterol ester uptake in the liver, this receptor is highly expressed on foam cells in human and mouse atherosclerotic lesions, where it may influence lesion development by affecting both the uptake of lipoproteins and the efflux of cholesterol to HDL [44]. The other class D SRs, CD68, and its murine homolog macrosialin are predominantly expressed in late endosomes and lysosomes of macrophages and may play a role in endolysosomal processing for oxLDL [45] (Figure 3).

### 2.5. ATP-Binding Cassette Transporters, Subset A and G (ABCA and ABCG)

 Free cholesterol released from lysosomes and rehydrolysed cholesteryl ester droplets can also traffic to the plasma membrane and thus be available for efflux out of the cells [46]. Cholesterol efflux is thought to be a major process involved in plaque regression when hypercholesterolemia is reversed. The mechanisms and exact route of cholesterol transport to the plasma membrane are not fully known, although Golgi-to-plasma membrane vesicular transport may be involved [47]. Once at the plasma membrane, cholesterol is transferred to the outer leaflet, where it is removed from cells by ABCA1- and ABCG1-mediated transport to apolipoprotein A1 and HDL, respectively, or by “passive diffusion” to cholesterol-poor HDL [48]. As predicted, genetic deficiencies of ABCA or ABCG could account for enhanced inflammation in atherosclerosis, especially after treatment with TLR ligands [49] and result in foam cell formation and further acceleration of atherosclerosis [50]. Extensive work* in vitro* and *in vivo* has focused on how sterol-regulated transcription factors, liver X receptors LXRa and LXRb (LXR), induce ABCA1 and ABCG1 and promote regression of foam cell lesions through this and other mechanisms [51]. Free cholesterol (FC) within macrophages has recently been proposed as an initiator of a proinflammatory signalling response in developing atherosclerotic lesions [52]. Oxysterols, from FC phagocytosis, are LXR agonists and increase reverse cholesterol transport (RCT) from macrophages by increasing expression of macrophage apolipoprotein E (apo E) and the cholesterol efflux transporters ABCA1 and ABCG1. This is likely an important part of the mechanism for LXR-dependent protection from atherosclerosis because these effects are not observed in LXR knockout mice [53]. Because accumulation of FC within macrophages at sites of atherosclerotic lesions converts them into foam cells [54] by stimulating RCT, LXR reduces foam cell formation and lesion cholesterol content directly (Figure 3). As a therapeutic strategy to promote lesion regression, investigators have attempted to enhance macrophage cholesterol efflux by increasing HDL or HDL-like particles or by increasing ABC transporters [48]. Though no drugs have yet been approved for this purpose, this approach continues to be a major focus of cardiovascular drug discovery.

### 2.6. Apoptosis of Macrophages in Early Atherosclerotic Lesion

The mechanism and role of macrophage apoptosis in early lesions are still not well understood. It is difficult to detect macrophage apoptosis in early lesions because apoptotic cells are rapidly cleared by the adjacent macrophages through phagocytosis (known as efferocytosis), which will be described later in the section of advanced progression in atherosclerosis (Figure 1). Several studies determined the effect of apoptosis on the progression of atherosclerosis. *Ldlr*–/– mice develop high levels of LDL when placed on a high-fat diet, because their hepatocytes lack LDL receptors and thus cannot efficiently eliminate the atherogenic LDL particles from the blood [55]. In *Ldlr*–/– mice in which bone marrow derived cells, including regional macrophages, are deficient of the proapoptotic protein Bax, the aortic lesions showed decreased macrophages apoptosis. Additionally, these lesions were larger and more macrophage-rich [56]. Conversely, *Ldlr*−/− mice, which lack the prosurvival protein AIM, showed an increase in apoptosis of early regional macrophages and developed smaller atherosclerotic lesions [57]. Thus, the apoptosis of the early regional macrophages is associated with lesion size and plaque progression. Deficiency of phospholipase C*β*3 resulted in enhanced sensitivity of newly recruited macrophages to oxLDL-induced apoptosis in early lesions, accompanied by a concomitant decrease in atherosclerosis [58]. Because knocking out phospholipase C*β*3 does not appear to change the mouse phenotype, this may be an attractive target to modulate macrophage apoptosis.

## 3. Advanced Progression in Atherosclerosis

**Figure 4:**
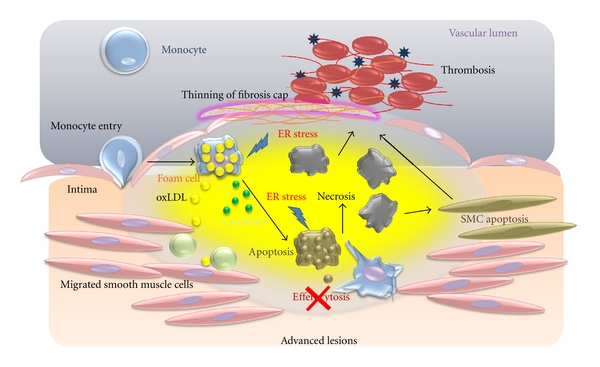
Cellular interactions with macrophages in an advanced lesion. Foam cells accumulate in the intima and undergo apoptosis that is triggered by cytokines. Efferocytes do not function properly, and apoptotic cells secondarily become necrotic cells, contributing to the formation of a necrotic core. Necrosis of macrophages and SMCs decrease collagen synthesis to diminish the collagen content of the fibrous cap, triggering rupture and thrombosis.

Macrophages in advanced atherosclerosis contribute to the plaque morphology, thinning the fibrous cap, and necrotic core, which can lead to increased pro-inflammatory responses and further apoptotic signals for SMCs, ECs, and leukocytes within the plaques [59]. The vulnerable plaque is prone to rupture and induction of thrombosis. In autopsy specimens containing atherosclerotic lesions, rupture sites were responsible for the acute vascular events [60]. The rupture sites, which are located on the shoulder of raised lesions, are almost always in the areas close to plaques' necrotic cores, and are associated with the thinning of fibrous caps. One of the most important questions in atherosclerosis is how macrophages contribute to this advancement in plaque progression (Figure 4).

 Macrophages decrease intimal myofibroblast-like SMCs and degradation of collagens [61] (Figure 4). *In vitro* data show that macrophages can trigger apoptosis of SMCs by activating the Fas apoptotic pathway and secreting proapoptotic TNF*α* and nitric oxide [62]. Macrophages may also decrease collagen synthesis in intimal SMCs through the secretion of macrophage-derived matrix metalloproteinases (MMPs) to decrease collagen synthesis [63]. MMPs may also be involved in thinning of the fibrous cap. In a study that attempted to look directly at plaque disruption, macrophage overexpression of MMP-9 had little effect on *Apoe*−/− mice due to a lack of MMP activation in plaques, but the overexpression of a constitutively active mutant form of MMP-9 resulted in plaque fissures [64]. Further details about TNF*α* and MMPs are discussed below in the biomarkers section.

### 3.1. Plaque Necrosis and Macrophage Death in Advanced Atherosclerotic Lesions

Plaque necrosis contributes to inflammation, thrombosis, plaque breakdown, and physical stress on the fibrous cap [65]. Necrotic cores arise from the combination of apoptosis of macrophages and the phagocytic clearance of the apoptotic cells in advanced plaques [18]. There is emerging evidence that SR-A plays different roles in early and advanced atherosclerotic lesions. As we described previously, SR-A has the protective function in early lesions. However, in advanced atherosclerotic lesions, in which macrophage cell death leads to necrotic core formation and plaque destabilisation, SR-A may have important roles in both the induction of apoptosis and clearance of these dying cells. In hypercholesterolemia, macrophage pathways for metabolising modified lipoproteins are thought to be overwhelmed, leading to a toxic accumulation of free cholesterol in the cells that result in the endoplasmic reticular stress. In this setting, the engagement of SR-A pathways by modified lipoproteins or fucoidan triggers apoptotic cell death, indicating that the SR-A signalling contributes to macrophage death and necrotic core formation [66]. However, this proatherosclerotic role is also balanced by the ability of SR-A to recognise and clear apoptotic cells in a nonphlogistic manner. These additional functions of SR-A must be considered when proposing therapies to inhibit this pathway. Longer-term studies of SR-A manipulation will be required to determine the impact of this receptor at later stages of atherosclerosis. 

A number of processes in advanced lesions may trigger macrophage death, and it is almost certain that a combination of factors and processes plays a role *in vivo*. A potential role for these processes is supported primarily by studies with cultured macrophages. The endoplasmic reticulum (ER) stress, primarily established by Tabas laboratory, may lead significantly to macrophage apoptosis and generation of necrotic core [67]. The high levels of ER stresses, such as intracellular oxysterols, lead to activate the unfolded protein response (UPR) pathway, which increases the expression of a proapoptotic protein, called CEBP-homologous protein (CHOP) [68]. The elevation of CHOP can trigger macrophage apoptosis by several mechanisms, but recent work shows a specific apoptotic mechanism involving calcium channel activity in the ER lumen [69]. Most importantly, a deficiency of CHOP in the models of advanced atherosclerosis suppresses advanced lesions due to macrophage apoptosis and plaque necrosis. Calcium released from the ER can trigger apoptosis through excess uptake into mitochondria that activates calcium/calmodulin-dependent protein kinase II (CaMKII), which, in turn, promotes cell apoptosis by activating both Fas death receptor and mitochondrial membrane permeabilization [69]. Another system may provide subtle ER stress, in which a “second hit” is needed to trigger apoptosis [70] (Figure 3). In this system, ER stress and macrophage apoptosis are induced by low-dose ER stressors including thapsigargin or 7-ketocholesterol, and combination of pattern recognition receptors activation as the “second hits”, each of which is unable to induce apoptosis by themselves [70] (Figure 3). An example of PRR activation is activators of SR-A and TLR 4, such as oxLDL. The other experiment demonstrated that activators of CD36 and TLR2/6, such as oxLDL and oxidized PLs (oxPL), can enhance the apoptosis pathways [67] (Figure 3). The role of SR-A and CD36 as the “second hits” for ER stress-induced apoptosis was demonstrated by a mouse model in which these receptors were targeted, with a result that apoptosis of advanced regional macrophages and plaque necrosis were deceased [71]. In humans, advanced plaques show similar results to those seen in mice. Autopsy specimens from human coronary arteries with heart disease showed a correlation with expression of markers of the UPR, including CHOP, apoptosis, and advanced plaque stage [72].

 Notably, macrophage apoptosis does not trigger plaque necrosis. Plaque necrosis and rupture occurs when apoptotic cells are not cleared sufficiently. Tabas called this phenomenon efferocytosis, which describes the phagocytic clearance of apoptotic cells [18]. Efferocytosis in early lesions prevents cellular necrosis and triggers anti-inflammatory pathways through TGF-*β* and the activation of the NF-*κ*B cell survival pathway (Figure 1) [73]. However, how efferocytosis becomes defective in advanced lesions is still unknown. It is assumed that the efferocytosis does not occur in advanced lesions, resulting in defective anti-inflammatory signalling (Figure 4) [18].

## 4. Biomarkers as Risk Factors Associated with Macrophages in Atherosclerosis

**Figure 5:**
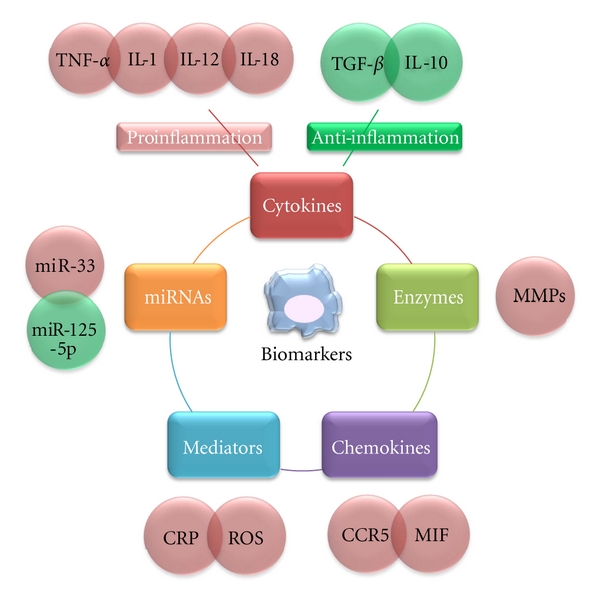
Pro- and anti-inflammatory biomarkers and microRNAs related to macrophages. All of these inflammatory markers and mediators, released at different stages of progression in atherosclerosis, can enter the circulation, affecting the prognosis of patients with atherosclerosis.

Given the new understanding of inflammation in atherosclerosis and their central role of macrophages, inflammatory biomarkers for disease progression in atherosclerosis should be independent of cholesterol and regulators of blood. In this regard, we will discuss biomarkers related to macrophages and inflammation in atherosclerosis (Figure 5). As our understanding of the biology of atherothrombosis has improved [74], several studies have evaluated a series of candidate biomarkers of inflammation, oxidative stress, and thrombosis as potential clinical tools to improve the prediction of risk in atherosclerosis [75, 76]. Although there are hundreds of papers that discuss the important functions of many mediators of atherosclerosis, the distinction between biomarkers versus mediators of disease has proven quite confusing. As discussed above, a particular molecule may participate clearly in a pathogenic pathway but not serve as an effective biomarker. For example, soluble VCAM-1 is not a useful indicator of risk of future myocardial infarction in apparently healthy men [77]. However, researchers have demonstrated that VCAM-1 is essential for the initiation of an atherosclerotic lesion [78].

### 4.1. Involvement of Cytokines Secreted by Macrophages in Atherosclerosis

#### 4.1.1. Tumour Necrosis Factor-*α* (TNF-*α*)

TNF-*α* regulates a number of critical cell functions including cell proliferation, survival, differentiation, and apoptosis. Macrophages produce TNF-*α* induced by TLRs and are also highly responsive to TNF-*α* through the TNF receptor (TNFR) [79, 80]. One of the various functions is a pivotal role in orchestrating the production of a pro-inflammatory cytokine cascade. TNF-*α* is thus considered to be a “master regulator” of pro-inflammatory cytokine production [81]. TNF-*α*-deficient *Apoe*−/− mice show a reduction in lesion formation, with a concomitant decrease in VCAM-1 and ICAM-1 expression, which are important for monocyte rolling on endothelial cells as mentioned previously [82]. In contrast, mice deficient in the TNF-*α* receptor (TNFR) develop larger lesions than control mice [83]. In addition to these roles, Witsell and Schook [84] demonstrated that TNF-*α* has macrophage differentiation capabilities. TNF-*α* gene transcripts are expressed during differentiation of bone-marrow-derived macrophages. TNF-*α* affects the development of atherosclerosis at the fatty streak stage, and cleavage of TNF is an important step in activating the proatherogenic properties of TNF-*α* [85].

#### 4.1.2. Interleukin 1 (IL-1)

 IL-1 stimulation initiates leukocyte adhesion to ECs for macrophage transmigration and contributes to slowly progressing inflammatory processes that take place in atherosclerosis [86]. Studies involving blocking IL-1ra antibodies in *Apoe*−/− mice and with *Ldlr*−/− transgenic mice that overexpress IL-1 or that have a deficiency in IL-1*β* clearly show that IL-1 is involved in atherogenesis [87]. Yet, although the circulating levels of IL-1, even in severe inflammatory diseases, are undetectable, the availability of anti-IL-1 antibodies will likely be very useful in the future [86].

#### 4.1.3. IL-12

IL-12 is a key Th1 cytokine that is produced mainly by plaque macrophages and stimulates the proliferation and differentiation of NK cells and T cells. IL-12 is detected in the aortas of *Apoe*–/– mice, and the administration of IL-12 results in enhanced lesion size in *Apoe*−/− recipients [88]. IL-12 p40-deficient *Il12b*−/−*Apoe*−/− mice have a 52% reduction of the plaque area at 30 weeks, but not at 45 weeks of age [89]. T lymphocyte recruitment into the intima was accelerated in early and advanced atherosclerotic lesions [16]. Most of the T cells are TCR*αβ* CD4+ cells with an activated phenotype, and a few express CD8+ or TCR*γδ* [90]. Interestingly, analysing CD4+ T cells showed that IL-12 upregulates CCR5 expression, chemotaxis, and transendothelial migration toward CCL5 through IL-12 receptors [91].

#### 4.1.4. IL-18

 IL-18 is produced by macrophages and administration of IL-18 antibodies accelerates development of atherosclerotic lesions in Apoe−/− mice. IL-18 seems to enhance atherosclerosis by increasing IFN-*γ* [92]. Although IL-18 is not currently considered a useful tool for the presence of subclinical atherosclerosis in general population [93], the AtheroGene Study indicates that high serum concentrations of IL-18 likely cause cardiovascular death in patients with coronary artery disease [94].

#### 4.1.5. Soluble CD40 Ligand (CD40L)

Macrophages, T lymphocytes, ECs, SMCs, and DCs express CD40L, whereas CD40 is found on macrophages, ECs, and SMCs from atherosclerosis-prone vessels [9]. The interaction of CD40 with CD40L plays a significant role in thrombosis, but it also contributes to modulation of the immune response in plaques. Treatment with antibodies against CD40L reduces atherosclerosis in *Ldlr*−/− mice, with a concomitant decrease in macrophages and T cells and a reduction in VCAM-1 expression [95]. Further experiments using *Cd40lg*−/−*Apoe*−/− mice have demonstrated a proatherogenic role for CD40L in advanced atherosclerosis by promoting lipid core formation and plaque destabilisation [96]. Because preanalytical sampling conditions critically influence the soluble CD40L concentration, only plasma samples are appropriate for CD40L measurement [97]. Although CD40L is critical for the development of advanced lesions in animal experiments, the Dallas Heart Study suggests that CD40L is not identified in subclinical atherosclerosis in the general population [98]. However, high concentrations of CD40L are associated with increased vascular risk in healthy women according the results of the Women's Health Study [99].

### 4.2. Anti-Inflammation Factors

#### 4.2.1. IL-10

 IL-10, which is derived from monocytes and macrophages, is an important anti-inflammatory regulator for the development of advanced atherosclerosis. As expected, IL-10-deficient mice showed a decreased amount of collagen, induced by IFN-*γ* production in the atherosclerotic vessels [100]. Studies with *Il10*−/−*Apoe*−/− mice confirmed the atheroprotective properties of IL-10 in early stage atherosclerosis and showed that IL-10 promotes the stability of advanced plaques [101]. IL-10 is not a prognostic marker for cardiovascular diseases. Although, it is possible to test serum concentrations of IL-10, we anticipate future studies on the involvement of this marker.

#### 4.2.2. TGF-*β*

Several cell types, including macrophages, produce TGF-*β*. Studies with animal models suggest that local (rather than systemic) alterations in TGF-*β* activity may be important during atherogenesis and that TGF-*β* levels in tissues may be more informative than those in blood [102]. *Apoe*−/− mice that express a dominant-negative form of TGF-*β* receptor II in T cells clearly demonstrated substantial roles for TGF-*β* in controlling the Th1 response in atherosclerosis [103]. Several studies suggest that TGF-*β* levels are reduced at sites of atherosclerotic plaque development. Introducing blocking antibodies against TGF-*β* or treatment with soluble TGF-*β* receptor II accelerates atherosclerosis with a significant loss of collagen content [87]. Although a direct measure of the ligand is technically demanding, associations between heart disease and genetic polymorphisms that are known to modulate ligand production might prove more accessible. Furthermore, such associations would support a causal relationship between altered TGF-*β* production and diseases [102]. A number of studies have examined the association between these polymorphisms and cardiovascular disease status. A large study of more than 6000 individuals who were involved in the Rotterdam study found an association between TGF-*β*1 polymorphisms and stroke (another pathology associated with plaque rupture, but in a different vascular field) [104]. Recently, using autopsy sections of atherosclerosis in a Japanese population, Oda et al. observed a significant association between atherosclerosis and the only TGF-*β*1 gene polymorphism, at least in some artery fields [105]. Taken together, these studies suggest that decreasing production of TGF-*β*1 ligands might favour unstable lesion phenotypes without affecting the plaque burden, once again highlighting the need to carefully select the cardiovascular endpoint under study.

### 4.3. Chemokines Produced by Macrophages in Atherosclerosis

#### 4.3.1. Chemokine Receptor CCR5

CCR5 was initially known as a coreceptor on macrophages for HIV infection. However, evidence now supports a role for CCR5 and its ligands CCL3 (MIP-1a), CCL4 (MIP-1b), and CCL5 (RANTES) in the initiation and progression of atherosclerosis [106]. Although there is no CCR5 in normal coronary arteries, CCR5 immunoreactivity is detected in atherosclerotic lesions, suggesting colocalisation of VSMC with macrophages [107]. It has been suggested that CCR5 may be more important in the later stages of plaque development [108]. A recent study found more than 50% reduction in the size of plaque lesions in the aortic root and the abdominal aorta of *Apoe*−/−*Ccr5*−/− mice and fewer macrophages in lesions compared with *Apoe*−/− mice [109]. The combined inhibition of three chemokine–receptor systems, MCP-1 (CCL2)/CCR2, fractalkine (CX3CL1)/CX3CR1, and CCL5/CCR5, was reported to abolish development of atherosclerosis in an* Apoe*−/− mouse model [110], supporting nonredundancy of these chemokines with regard to monocyte mobilisation in atherosclerosis. Compared with chemokine receptors, the ligands CCL3, 4, and 5 seem to be better choices for biomarkers in atherosclerosis because it is possible to test their mRNA levels in circulating leukocytes. The role of CCL3 and CCL4 acting on CCR5 in atherogenesis is less well defined, but these chemokines also appear to be important in atheroma progression and inflammatory cell recruitment into plaques [111]. In particular, findings from animal models indicate that CCL5 plays a greater role in the development of atherosclerotic plaque than other CCR5 ligands [112].

### 4.4. Macrophage Migration Inhibitory Factor (MIF)

MIF is produced by macrophages in early and advanced atherosclerotic lesions. The role of MIF with respect to inflammatory cell recruitment in atherosclerotic plaque progression has been described [113]. A study of *Mif*−/−*Ldlr*−/− mice suggested that MIF is involved in atherosclerosis through the regulation of lipid deposition, protease expression, and intimal thickening [114]. Because MIF can be readily measured in plasma and other tissue fluids in different disease states [115], the different roles of MIF as a biomarker in pathogenesis and progression of atherosclerosis are an important area of inquiry.

### 4.5. Inflammation-Regulating Enzymes: Matrix Metalloproteinases (MMPs)

Macrophage-derived MMPs are involved in the thinning of the fibrous cap [116]. MMPs are a family of protease-activated enzymes that degrade extracellular matrix (ECM) proteins. The regulation of MMPs is complex; once activated, an MMP can activate others. Studies showing a temporal and spatial correlation between the presence of macrophages in shoulder plaque regions, thinning of the fibrous cap in these regions, MMP-2 and MMP-9, have stimulated great interests in the potential roles of MMPs in plaque rupture [117]. According to the follow-up data, plasma MMP-9 during acute coronary syndromes is increased two to three times compared with controls [118]. However, whether MMP-9 becomes the independent prognostic marker still requires further and large-scale research. A targeted approach that inhibits MMPs has already been considered [119].

### 4.6. Proinflammatory Mediators Associated with Macrophage: C-Reactive Protein (CRP)

Multiple large-scale studies demonstrate that CRP strongly and independently predicts adverse cardiovascular events, including myocardial infarction, ischemic stroke, and sudden cardiac death because of atherosclerosis [120, 121]. However, these mechanisms have not been comprehensively identified. CRP is found close to LDL and macrophages within atherosclerotic plaques. Recently, several reports demonstrated that CRP could modulate endothelial functions and leukocyte activities. CRP also induces the production of IL-1*α*, IL-1*β*, IL-6, CXCL1, and CXCL8 by human monocytes *in vitro*. In contrast to these proinflammatory properties, CRP also displays anti-inflammatory effects through the upregulation of liver X receptor-*α* [122]. CRP binds to minimally modified (mm) LDL to prevent the foam cell formation from macrophages [123]. Based on animal experiments and the cardiovascular risk stratification in primary prevention populations, the Centers for Disease Control and Prevention and the American Heart Association assigned CRP as an independent marker of cardiovascular risk. The recommended cut-off points in clinical practice are 1 mg/L for low-risk and 3 mg/L for high-risk individuals [124].

### 4.7. Superoxide Production: Reactive Oxygen Species (ROS)

Extensive ROS has been implicated in atherosclerosis by inducing the chronic activation of vascular endothelium and components of immune systems. It has been demonstrated that superoxide production from both macrophages and vascular cells plays a critical role in atherogenesis [125]. When ROS production exceeds the scavenging capacity of cellular antioxidant systems, the resulting oxidative stresses damage lipids, membranes, proteins, and DNAs.

### 4.8. Emerging Future Biomarkers: MicroRNAs (miRNAs)

miRNAs are highly conserved single-stranded noncoding small RNAs that control cellular functions by either degrading mRNAs or inhibiting their translation [126]. The involvement of miRNAs in different aspects of cardiovascular diseases has emerged as an important research field. The dysregulation of many individual miRNAs has been linked to the development and progression of cardiovascular diseases. The forced expression or suppression of a single miRNA is enough to cause or alleviate pathological changes. The roles of miRNAs in the pathogenesis of heart and vascular diseases suggest the possibility of using miRNAs as a potential diagnostic biomarker and/or therapeutic target for cardiovascular diseases [127].

As previously discussed, a critical step in the development of chronic inflammatory atherosclerotic diseases is the migration of circulating monocytes into the subendothelial space and their differentiation into macrophages. A recent study showed that miR-125a-5p mediates lipid uptake and decreases the secretion of some inflammatory cytokines, including IL-2, IL-6, TNF-*α*, and TGF-*β* from oxLDL-stimulated monocyte-derived macrophages [128]. The target gene of miR-125a-5p has been found to be ORP9, which has diverse roles in the regulation of lipid metabolism, including vesicle transport, and cell cycle regulation and differentiation [129]. miR-33 appears to regulate both HDL biogenesis in the liver and cellular cholesterol efflux [130]. miR-33 is an intronic miRNA located within the gene encoding sterol-regulatory element-binding factor-2, a transcriptional regulator of cholesterol synthesis. miR-33 modulates the expression of genes that are involved in cellular cholesterol transport. It appears to be regulated by dietary cholesterol* in vivo* and have several roles in cholesterol homeostasis [131]. miR-33 targets the 3′ UTR of ABCA1 in mouse peritoneal macrophages and human cells [131, 132], resulting in reduced atherogenic cholesterol efflux to apolipoprotein A1. Similarly, in a mouse model, the lentiviral delivery of miR-33 represses ABCA1 expression in the liver, leading to a reduction in circulating HDL levels, whereas mice expressing anti-miR-33 demonstrate increased plasma HDL levels [132]. Clearly, miR-33 is a promising target for the treatment of abnormalities in lipoprotein metabolism that frequently contributes to atherosclerosis.

## 5. Conclusion

The accumulation of macrophages laden with cholesterol in the vascular intima is the hallmark of fatty plaque formation in atherosclerosis. Understanding the mechanisms involving macrophages is critical for the prognosis, diagnosis, and treatment of atherosclerosis. However, because most papers cited in this paper show data from cultured macrophages and animal models, these data may not completely reflect the process in human diseases. As noted by Rosenfeld et al., mouse atherosclerosis is not a good model for true plaque rupture or thrombosis [133]. In contrast, some papers on atherosclerosis emphasise the fact that many genes involved in macrophages have “major and critical” functions for plaques, which complicates the process of determining useful biomarkers for atherosclerosis. Human genetic studies and mechanism-based clinical trials should be performed in the future.
